# Psp Stress Response Proteins Form a Complex with Mislocalized Secretins in the *Yersinia enterocolitica* Cytoplasmic Membrane

**DOI:** 10.1128/mBio.01088-17

**Published:** 2017-09-12

**Authors:** Disha Srivastava, Amal Moumene, Josué Flores-Kim, Andrew J. Darwin

**Affiliations:** Department of Microbiology, New York University School of Medicine, New York, New York, USA; University of Chicago

**Keywords:** secretin, *Yersinia*, protein interactions, stress response

## Abstract

The bacterial phage shock protein system (Psp) is a conserved extracytoplasmic stress response that is essential for the virulence of some pathogens, including *Yersinia enterocolitica*. It is induced by events that can compromise inner membrane (IM) integrity, including the mislocalization of outer membrane pore-forming proteins called secretins. In the absence of the Psp system, secretin mislocalization permeabilizes the IM and causes rapid cell death. The Psp proteins PspB and PspC form an integral IM complex with two independent roles. First, the PspBC complex is required to activate the Psp response in response to some inducing triggers, including a mislocalized secretin. Second, PspBC are sufficient to counteract mislocalized secretin toxicity. Remarkably, secretin mislocalization into the IM induces *psp* gene expression without significantly affecting the expression of any other genes. Furthermore, *psp* null strains are killed by mislocalized secretins, whereas no other null mutants have been found to share this specific secretin sensitivity. This suggests an exquisitely specific relationship between secretins and the Psp system, but there has been no mechanism described to explain this. In this study, we addressed this deficiency by using a coimmunoprecipitation approach to show that the Psp proteins form a specific complex with mislocalized secretins in the *Y. enterocolitica* IM. Importantly, analysis of different secretin mutant proteins also revealed that this interaction is absolutely dependent on a secretin adopting a multimeric state. Therefore, the Psp system has evolved with the ability to detect and detoxify dangerous secretin multimers while ignoring the presence of innocuous monomers.

## IMPORTANCE

The phage shock protein (Psp) response has been linked to important phenotypes in diverse bacteria, including those related to antibiotic resistance, biofilm formation, and virulence. This has generated widespread interest in understanding various aspects of its function. Outer membrane secretin proteins are essential components of export systems required for the virulence of many bacterial pathogens. However, secretins can mislocalize into the inner membrane, and this induces the Psp response in a highly specific manner and kills Psp-defective strains with similar specificity. There has been no mechanism described to explain this exquisitely specific relationship between secretins and the Psp system. Therefore, this study provides a critical advance by discovering that Psp effector proteins form a complex with secretins in the *Yersinia enterocolitica* inner membrane. Remarkably, this interaction is absolutely dependent on a secretin adopting its multimeric state. Therefore, the Psp system detects and detoxifies dangerous secretin multimers, while ignoring the presence of innocuous secretin monomers.

## INTRODUCTION

The bacterial cell envelope serves many essential functions, including its role as a permeability barrier and in maintaining cell shape (1). However, it is vulnerable to adverse conditions and damaging molecules, which are collectively referred to as extracytoplasmic or envelope stress. Bacteria counteract these threats with envelope stress responses (ESRs), of which there are several well-characterized examples (2–4). ESRs detect deleterious conditions within the cell envelope and transduce a signal to the cytoplasm in order to elicit changes in gene expression. Some ESRs, such as the RpoE, Cpx, and Rcs responses, cause extensive changes in Gram-negative bacteria, altering the expression of numerous genes to impact various cell envelope functions (2, 5, 6). In contrast, the phage shock protein (Psp) ESR causes a highly restricted transcriptional response (7, 8). The Psp system has been studied extensively in *Escherichia coli* and also in the human pathogen *Yersinia enterocolitica*, where it is essential for virulence (9). It can be induced by a variety of conditions, including extreme temperatures, osmolarity, and ethanol concentrations, all of which could have a negative impact on the inner membrane (IM) (7, 8).

The Psp response was discovered in *E. coli* when the level of a bacterial protein later named PspA was massively increased during filamentous phage f1 infection (10). In *Y. enterocolitica*, the Psp system is encoded by the *pspF-pspABCDycjXF* locus and the unlinked gene *pspG* (9, 11). However, the PspA, PspB, PspC, and PspF proteins are considered its core components required for regulation and stress tolerance. PspF is a transcription factor that binds to the *pspA* and *pspG* control regions and activates their σ^54^-dependent promoters (11, 12). PspA can interact with PspF and inhibit it (13). PspB and PspC form an integral IM complex that switches from off to on states when an inducing signal is encountered (14–17). In the on state, the C-terminal cytoplasmic domain of PspC is able to bind to PspA, which releases PspF to activate transcription (14, 18).

In contrast to extreme environmental conditions, outer membrane (OM) pore-forming proteins known as secretins are highly specific inducers of the Psp response when they mislocalize into the IM (19, 20). Secretins are key components of type II and type III secretion systems (T3SS), type IV pili, and filamentous bacteriophage exporters (21). It is the mislocalization of the filamentous phage f1-encoded pIV secretin into the IM that induces the *E. coli* Psp response during phage infection (10). Secretins assemble into multimers that normally form a pore in the OM through which the cargo of their export system is secreted. They are defined by a conserved C-terminal secretin domain which forms a ring-like structure in the multimer that is embedded in the OM (21). Their N-terminal regions project into the periplasm and are more variable, because they interact with components of their specific export system. Secretins have been characterized into several classes on the basis of their membrane targeting and oligomer assembly requirements (22). *In vivo*, some proteins can assist the targeting and assembly of secretins, and these have been classified as pilotins or other accessory proteins (22). Secretins have also been divided into those that can or cannot multimerize spontaneously, a property that is influenced by a conserved proline residue (23).

As mentioned above, secretin mislocalization into the IM induces the Psp response without significantly affecting the expression of any other genes (19, 20). Furthermore, Psp-defective strains are killed by mislocalized secretins, but a random screen in *Y. enterocolitica* did not identify any other null mutants that share this specific sensitivity to secretins (9, 20). Mislocalized secretins kill *psp* null strains by causing catastrophic IM permeability, and the PspB and PspC proteins alone can prevent this from happening (24, 25). Together, all of these observations suggest that there is a highly specific relationship between secretins and the Psp system, especially its PspB and PspC components. Therefore, it follows that there must be a molecular mechanism underlying this specificity. In this study, we identified such a mechanism by discovering that Psp effector proteins form a complex with secretins in the IM. Our data also reveal that this interaction depends on a secretin adopting its multimeric state, suggesting that the Psp system can distinguish dangerous multimers from innocuous monomers.

## RESULTS

### Coimmunoprecipitation of a complex containing Psp proteins and the YsaC secretin.

When the *Y. enterocolitica* Psp response is induced by secretin mislocalization, PspA, -B, and -C interact and colocalize close to the cell pole (18). Intriguingly, others have reported that when a secretin-mCherry secretin fusion protein was mislocalized into the *Y. enterocolitica* IM, it also formed clusters at the cell pole (26). These apparently similar locations of Psp proteins and a mislocalized secretin led us to hypothesize that they might interact with one another, which would offer a compelling mechanism for their highly specific relationship. Therefore, we used a coimmunoprecipitation (co-IP) approach to test this hypothesis.

**FIG 1:**
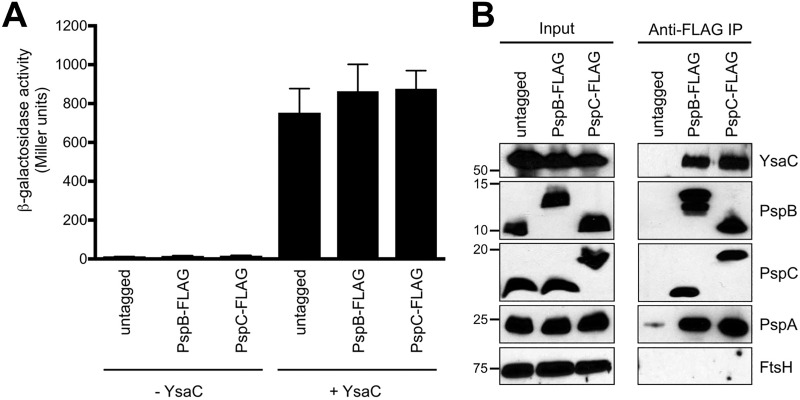
The YsaC secretin coimmunoprecipitates with Psp effector proteins. (A) Φ(*pspA-lacZ*) operon fusion expression. All strains had the chromosomal *pspA* operon controlled by the *tac* promoter and either *pspB*, *pspC*, or neither (untagged) modified to encode a C-terminal 3×FLAG epitope tag, as indicated. Strains also contained empty *araBp* expression plasmid pBAD33 (−YsaC) or a derivative encoding YsaC-His_6_ (+YsaC). Error bars indicate the positive standard deviations from the means. (B) Immunoblot analysis of input lysates and coimmunoprecipitates (anti-flag IP) derived from the +YsaC strains used in the experiment summarized in panel A. PspA, PspB, PspC, and FtsH were detected with polyclonal antisera, and YsaC-His_6_ was detected with anti-His_6_ monoclonal antibody. Approximate positions of molecular mass marker proteins (in kilodaltons) are indicated on the left.

We focused on using PspB or PspC as the bait, because these two proteins are essential for secretin-dependent induction of the Psp response, and they are sufficient to prevent mislocalized secretins from killing cells (24, 25). To facilitate the co-IPs, a sequence encoding the 3×FLAG epitope (here referred to as FLAG) was fused to the end of the *pspB* or *pspC* chromosomal genes. We also used strains described previously (25, 27) in which chromosomal *pspA* operon expression is controlled by the *tac* promoter. Basal *tacp* expression leads to physiological levels of PspA, -B, and -C, which normally regulate Φ(*pspA-lacZ*) expression, but their protein levels remain constant, regardless of the induction status (important for later experiments comparing strains with or without Psp-inducing secretins). In the first experiment we compared strains in which PspB or PspC or neither protein was FLAG tagged, and all strains contained an *araBp-ysaC-his*_*6*_ expression plasmid to produce the YsaC secretin of the *Y. enterocolitica* Ysa-Ysp T3SS (a potent Psp inducer [24]). Analysis of Φ(*pspA-lacZ*) operon fusion expression showed that the FLAG tags did not compromise the regulatory functions of PspB or PspC and that YsaC-His_6_ induced the Psp response (Fig. 1A). Proteins were isolated from solubilized membrane lysates of these strains by immunoprecipitation with anti-FLAG monoclonal antibody under native conditions. Immunoprecipitates were separated by SDS-PAGE and analyzed by immunoblotting (Fig. 1B). As expected, PspC coimmunoprecipitated with PspB-FLAG, and PspB coimmunoprecipitated with PspC-FLAG, and we were also able to detect PspA in both cases (Fig. 1B). This is consistent with our established findings that PspB and PspC always interact *in vivo* and that PspA joins them when the Psp system is induced (14, 16, 18, 24). Therefore, these observations served as a control for the effectiveness of the co-IP. Importantly, YsaC also coimmunoprecipitated with the Psp protein complexes, which supports our hypothesis of an interaction between secretins and one or more Psp proteins (Fig. 1B). As a control, we tested for the abundant IM protein FtsH, but it was not present in any immunoprecipitates. Finally, in a strain in which neither PspB or PspC was FLAG tagged, YsaC was not present in the immunoprecipitates, showing that YsaC does not interact nonspecifically with FLAG antibodies or the protein A-sepharose to which they were attached (a trace amount of PspA was present, which is consistent with previous observations showing background binding of PspA to sepharose [28, 29]).

**FIG 2:**
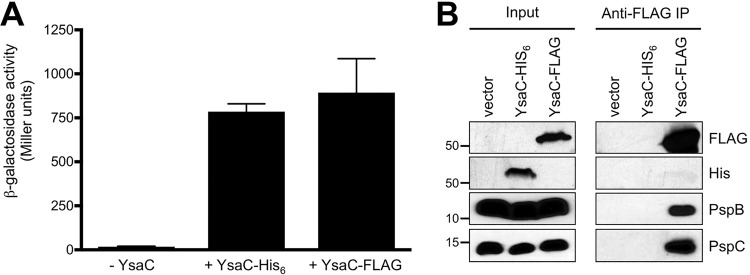
PspB and PspC coimmunoprecipitate with YsaC. (A) Φ(*pspA-lacZ*) operon fusion expression. Strains contained the chromosomal *pspA* operon controlled by the *tac* promoter and empty *araBp* expression plasmid pBAD33 (−YsaC) or the derivatives encoding YsaC-His_6_ or YsaC-FLAG as indicated. Error bars indicate the positive standard deviations from the means. (B) Immunoblot analysis of input lysates and coimmunoprecipitates (anti-FLAG IP) derived from the strains used in the experiment summarized in panel A. PspB and PspC were detected with polyclonal antisera, and YsaC-His_6_ and YsaC-FLAG were detected with anti-His_6_ or anti-FLAG monoclonal antibodies, respectively. Approximate positions of molecular mass marker proteins (in kilodaltons) are indicated on the left.

To further test the validity of our findings, we did a reverse co-IP in which we used YsaC as the bait rather than the prey. Proteins were isolated by anti-FLAG IP from strains producing YsaC-FLAG or YsaC-His_6_, both of which induced the Psp response to a similar extent (Fig. 2A). As expected, PspB and PspC were present in the immunoprecipitate from the strain producing YsaC-FLAG, but not from strains producing YsaC-His_6_ or not producing any YsaC (Fig. 2B).

### The interaction between PspBC and the YsaC secretin is specific.

**FIG 3:**
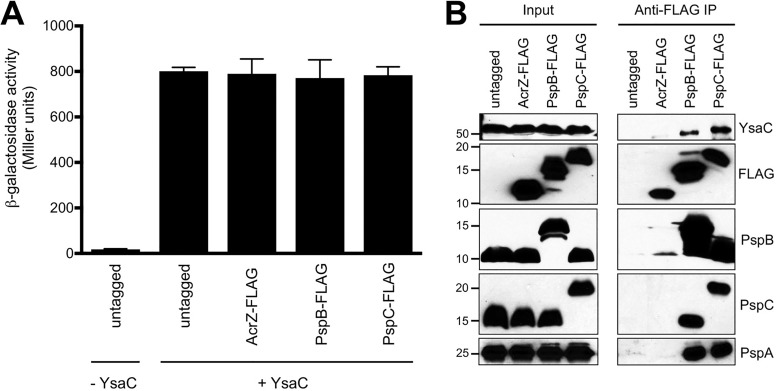
The YsaC secretin does not coimmunoprecipitate with the unrelated small inner membrane protein AcrZ. (A) Φ(*pspA-lacZ*) operon fusion expression. Strains contained the chromosomal *pspA* operon controlled by the *tac* promoter. The chromosomal *pspB* or *pspC* genes were modified to encode a C-terminal 3×FLAG epitope tag where indicated. A derivative of a strain where PspBC were not FLAG tagged contained a *rhaBp* expression plasmid encoding AcrZ-3×FLAG (AcrZ-FLAG; all other strains contained the empty *rhaBp* expression plasmid). Strains also contained empty *araBp* expression plasmid pBAD33 (−YsaC) or the derivative encoding YsaC-His_6_ (+YsaC). Error bars indicate the positive standard deviations from the means. (B) Immunoblot analysis of input lysates and coimmunoprecipitates (anti-FLAG IP) derived from the +YsaC strains used in the experiment summarized in panel A. PspA, PspB, and PspC were detected with polyclonal antisera, and YsaC-His_6_ was detected with anti-His_6_ monoclonal antibody. FLAG-tagged PspB and PspC were also detected with anti-FLAG monoclonal antibodies, as was AcrZ-FLAG. Approximate positions of molecular mass marker proteins (in kiladaltons) are indicated on the left.

The bait proteins PspB and PspC are both small integral IM proteins, and YsaC is overproduced to force its mislocalization into the IM. This raises the possibility that the overproduced YsaC secretin might coimmunoprecipitate with any small IM protein present at a similar concentration as PspB or PspC. To test this, we used AcrZ, which is a 49-amino-acid IM protein with a single transmembrane helix (by comparison, PspB is a 75-amino-acid protein with a single transmembrane helix). AcrZ interacts with the AcrAB-TolC multidrug efflux pump but it has no known relationship to the Psp response or to secretins (30). We repeated the original co-IP experiment (Fig. 1B), except that we included a derivative of the strain where PspBC were not FLAG tagged but contained a *rhaBp* expression plasmid encoding AcrZ-FLAG (all other strains contained the empty *rhaBp* expression plasmid). A rhamnose concentration of 0.005% (wt/vol) induced AcrZ-FLAG production to a similar level as PspB-FLAG and PspC-FLAG, as determined by detecting all three with the same FLAG antibody (Fig. 3B, input samples). Analysis of Φ(*pspA-lacZ*) expression confirmed that the presence of AcrZ-FLAG did not interfere with YsaC-dependent induction of the Psp response (Fig. 3A). Proteins were isolated by anti-FLAG IP as before, but YsaC did not coimmunoprecipitate with AcrZ-FLAG (Fig. 3B). These data support the conclusion that the coimmunoprecipitate of YsaC with PspB-FLAG or PspC-FLAG is specific.

### Other secretins that induce the Psp response also coimmunoprecipitate with PspB and PspC.

**FIG 4:**
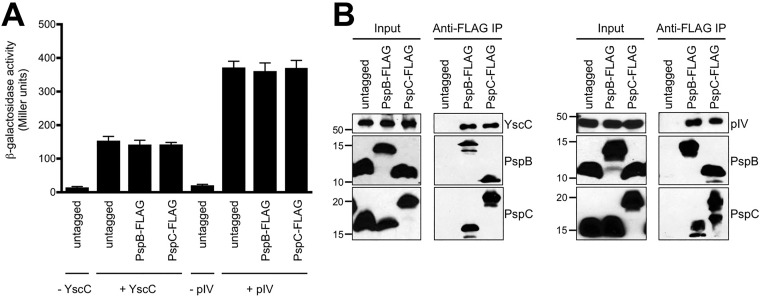
Other secretins that induce the Psp response also coimmunoprecipitate with PspB and PspC. (A) Φ(*pspA-lacZ*) operon fusion expression. Strains contained chromosomal *pspB* or *pspC* or neither (untagged) modified to encode a C-terminal 3×FLAG epitope tag, as indicated. Strains also contained empty *tacp* expression plasmid pVLT35 (−YscC), the pVLT35 derivative encoding YscC-His_6_ (+YscC), empty *araBp* expression plasmid pBAD18-Kan (−pIV), or the derivative encoding pIV-His_6_ (+ pIV). Error bars indicate the positive standard deviations from the means. (B) Immunoblot analysis of input lysates and coimmunoprecipitates (anti-FLAG IP) derived from the +YscC and +pIV strains used in the experiment summarized in panel A. PspB and PspC were detected with polyclonal antisera, and YscC-His_6_ and pIV-His_6_ were detected with anti-His_6_ monoclonal antibodies. Approximate positions of molecular mass marker proteins (in kilodaltons) are indicated on the left.

Different secretins, from both phage and various bacteria, induce the Psp response and are toxic to *psp* null strains (20, 24, 25). Therefore, we reasoned that if the YsaC secretin-Psp protein complex has functional significance, it should also occur with other Psp-inducing secretins. To investigate this, we used two additional secretins as prey proteins in co-IP experiments: the YscC secretin from the *Y. enterocolitica* virulence plasmid-encoded T3SS secretion system and the pIV secretin from *E. coli* filamentous phage f1. Production of YscC sufficient to impact the Psp system requires the use of a *tacp-yscC* expression plasmid rather than *araBp* (9). Therefore, for the YscC experiments we used strains where the *pspA* operon was natively expressed from its wild-type promoter rather than *tacp*. The co-IP experiment was repeated using expression plasmids encoding YscC-His_6_ or pIV-His_6_, and both were confirmed to induce Φ(*pspA-lacZ*) expression in the strains used to generate their co-IP samples (Fig. 4A). When proteins were isolated by anti-FLAG IP, both YscC and pIV coimmunoprecipitated with PspB-FLAG and also with PspC-FLAG, whereas neither of the secretins was present in immunoprecipitates from strains in which neither PspB nor PspC was FLAG tagged (Fig. 4B). These data suggest that the Psp proteins might be able to form a complex with any Psp-inducing secretin.

### A secretin that does not induce the Psp response does not coimmunoprecipitate with PspB and PspC.

**FIG 5:**
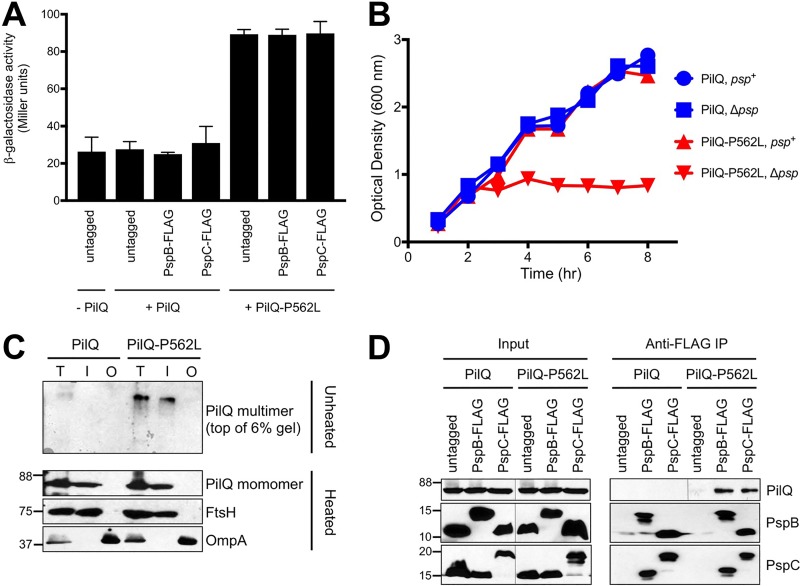
Induction of the Psp response, toxicity to a *psp* null strain, and interaction with Psp proteins all depend on the ability of the PilQ secretin to form multimers. (A) Φ(*pspA-lacZ*) operon fusion expression. Strains contained chromosomal *pspB* or *pspC* or neither (untagged) modified to encode a C-terminal 3×FLAG epitope tag as indicated. Strains also contained empty *tacp* expression plasmid pVLT35 (−PilQ) or derivatives encoding PilQ-His_6_ (+PilQ) or PilQ-P562L-His_6_ (+PilQ-P562L). Error bars indicate the positive standard deviations from the means. (B) Growth curves. *psp*+ (AJD3) and mutant Δ*psp* (AJD1171) strains containing the *pilQ* expression plasmids used for the experiment summarized in panel A were grown in medium containing 300 µM IPTG. Optical density was measured hourly. (C) PilQ subcellular localization and multimer detection. Anti-His_6_, anti-FtsH (inner membrane control), and anti-OmpA (outer membrane control) immunoblot analysis of subcellular fractions from the +PilQ and +PilQ-P562L-His_6_ strains used in the experiments summarized in panel A. T, total membrane fraction; I, inner membrane fraction; O, outer membrane fraction. Approximate positions of molecular mass marker proteins (in kilodaltons) are indicated on the left. (D) Immunoblot analysis of input lysates and coimmunoprecipitates (anti-FLAG IP) derived from the +PilQ strains used in the experiment summarized in panel A. PspB and PspC were detected with polyclonal antisera, and PilQ-His_6_ and PilQ-P562L-His_6_ were detected with anti-His_6_ monoclonal antibodies. Approximate positions of molecular mass marker proteins (in kilodaltons) are indicated on the left. Immunoblots were done simultaneously, but some parts were assembled by joining cropped sections to show the desired sample order (indicated by thin lines).

When the PilQ secretin from the type IV pilus of *Pseudomonas aeruginosa* is produced without its pilotin (PilF), it mislocalizes into the *P. aeruginosa* IM and cannot form multimers (31). We previously showed that PilQ produced in *Y. enterocolitica* does not induce the Psp response and is not toxic to a *psp* null strain (25). If secretin-Psp protein complexes have functional significance, we hypothesized that the PilQ secretin should not form a complex with the Psp proteins in *Y. enterocolitica*, because it has no impact on the Psp system. We tested this by using PilQ-His_6_ as the prey in our *Y. enterocolitica* co-IP experiments. For maximal PilQ-His_6_ production, we used a *tacp-pilQ* expression plasmid (pVLT35) and so as we did when studying YscC (Fig. 4), these experiments were done using strains in which the *pspA* operon was natively expressed. As expected, PilQ-His_6_ did not induce Φ(*pspA-lacZ*) expression, was not toxic to a *psp* null strain, and did not form SDS-resistant multimers, but it did localize to the IM fraction (Fig. 5). However, despite its presence in the IM, when proteins were isolated by anti-FLAG IP, PilQ did not coimmunoprecipitate with PspB-FLAG or with PspC-FLAG (Fig. 5D). These findings raise the intriguing possibility that the Psp proteins might recognize secretins only when they form multimers, rather than recognizing a conserved sequence feature found in all secretins, such as within their C-terminal secretin domains. We investigated this possibility in the next set of experiments.

### The Psp system can distinguish between monomeric and multimeric versions of the same secretin.

Some secretins can self-assemble into multimers *in vitro*, whereas others, such as PilQ, cannot (23). However, mutation of a conserved proline to leucine did allow the PilQ secretins from *Neisseria meningitidis* and *P. aeruginosa* to self-assemble *in vitro* (23). We reasoned that if this mutation in *P. aeruginosa* PilQ (P562L) would also allow it to multimerize *in vivo* in *Y. enterocolitica*, then it would provide an excellent tool to test the hypothesis that the Psp proteins recognize secretins only when they form multimers.

To investigate this, we constructed a derivative of the *tacp-pilQ* expression plasmid that encoded PilQ-P562L, and we tested its behavior in *Y. enterocolitica*. In contrast to wild-type PilQ, PilQ-P562L induced Φ(*pspA-lacZ*) expression, was toxic to a *psp* null strain, and formed SDS-resistant multimers in the IM fraction (Fig. 5). Thus, we now had two versions of PilQ that both mislocalized into the IM, but only one of them formed multimers. To test if this multimeric version of PilQ would form a complex with PspB and PspC, we used PilQ-P562L as the prey in our co-IP procedure. Unlike wild-type PilQ, PilQ-P562L did coimmunoprecipitate with PspB-FLAG and with PspC-FLAG, but it was not present in immunoprecipitates from strains in which neither PspB nor PspC was FLAG tagged (Fig. 5D). This supports our hypothesis that the Psp system interacts with secretins only when they form multimers, suggesting that one or more Psp proteins might recognize some feature of a secretin multimer or a consequence of multimer formation.

### Random identification of secretin mutants that cannot induce the Psp response.

All of the preceding data showed that Psp proteins form a complex with mislocalized secretins in the IM and suggested that some feature of a secretin multimer is required for this to occur. Next, we considered what the Psp system might recognize. One possibility is that it is some structural feature of a secretin multimer. Alternatively, specific contacts with amino acids of the secretin might be needed, with multimerization ensuring the correct number of these contacts for successful complex formation. In an attempt to investigate these possibilities, we designed a screen to identify secretin mutants that did not interact with the Psp proteins. We used the pIV secretin, because it is a relatively small target for random mutagenesis for a secretin (the monomers have a mass of 43.5 kDa).

We assumed that pIV mutants that do not interact with the Psp proteins would not induce Φ(*pspA-lacZ*) expression. Therefore, the insert of our pIV-His_6_ expression plasmid was randomly mutagenized by error-prone PCR and used to transform a *psp*^+^ Φ(*pspA-lacZ*) reporter strain (AJD977) (Table 1). Transformants were recovered on MacConkey agar at 26°C, in which enough pIV is produced to induce Φ(*pspA-lacZ*) expression and form red colonies but there is not enough to be toxic to a *psp* null strain (data not shown). Therefore, mutant colonies that were white/pink at 26°C were retained. At 37°C, pIV is produced at a higher level from the *araBp* expression plasmid that is toxic to a *psp* null strain (data not shown). Therefore, white/pink colonies from the 26°C plates were screened for growth at 37°C. Those that grew normally were discarded as likely null mutants, or nonmultimerizing mutants, which we suspected would not be toxic. Those that grew as poorly as a strain producing wild-type pIV were retained, with the reasoning that their failure to induce the higher Psp protein levels was responsible for the toxicity at 37°C. We selected 12 isolates that met these criteria, and another 5 (mutants 116, 117, 123, 132, and 195) (Table 2) that were toxic at 37°C but less toxic than wild-type pIV (data not shown). DNA sequence analysis revealed a broad spectrum of mutations, many of which were in the C-terminal secretin domain (Table 2).

**TABLE 1:** Strains and plasmids

Strain or plasmid	Genotype or description	Reference or source
*Y. enterocolitica* strains		
AJD3^a^	*ΔyenR* (r^−^ m^+^) Nal^r^	Lab collection
AJD977	*ΔyenR* (r^−^ m^+^) Δ*araGFB*::[Φ(*pspAp-lacZY*)]	49
AJD1171	*ΔyenR* (r^−^ m^+^) Δ(*pspF-ycjF*) *ΔpspG*	24
AJD3490	*ΔyenR* (r^−^ m^+^)::[*pspF*^+^] *ΔaraGFB*::[Φ(*pspAp-lacZY*)] *ΔpspF* Δ*pspAp*::(*lacI*^q^*-tacp*)	27
AJD4609	*ΔyenR* (r^−^ m^+^) Δ*araGFB*::Φ(*pspAp-lacZY*) Φ(*pspB-*3×FLAG)-hyb	This study
AJD4740	*ΔyenR* (R^−^M^+^) Δ*araGFB*:: Φ(*pspAp-lacZY*) Φ(*pspC-*3×FLAG)-hyb	This study
AJD4739	*ΔyenR* (r^−^ m^+^)::[*pspF*^+^] Δ*araGFB*::Φ(*pspAp-lacZY*) Δ*pspF ΔpspAp*::*lacI*^q^*-tacp* Φ(*pspC-*3×FLAG)-hyb	14
AJD4741	*ΔyenR* (r^−^ m^+^)::[*pspF*^+^] Δ*araGFB*::Φ(*pspAp-lacZY*) Δ*pspF ΔpspAp*::*lacI*^q^*-tacp* Φ(*pspB-*3×FLAG)-hyb	14
Plasmids		
pBAD18-Kan	Km^r^, Col E1, *ori araBp* expression vector	50
pBAD33	Cm^r^, p15A, *ori araBp* expression vector	50
pVLT35	Sm^r^ Sp^r^, RSF1010, *ori tacp* expression vector	51
pRE112	Cm^r^, R6K, *ori mob*^+^ (RP4) *sacB*^+^	52
pAJD935	*araBp-ysaC-his*_*6*_ in pBAD33	25
pAJD1806	*tacp-pilQ-his*_*6*_ in pVLT35	25
pAJD2144	Km^r^, pSC101 *ori*, *rhaBp* expression vector	18
pAJD2446	*tacp-pilQ*(*P562L*)*-his*_*6*_ in pVLT35	This study
pAJD2455	*tacp-yscC-his*_*6*_ in pVLT35	This study
pAJD2506	*araBp-gIV-his*_*6*_ in pBAD18-Kan	This study
pAJD2657	*rhaBp-acrZ-*3×FLAG in pAJD2144	This study
pAJD2779	*araBp-ysaC-*3×FLAG in pBAD33	This study

a AJD3 is a virulence plasmid-cured derivative of strain JB580v described by Kinder et al. (53). All other *Y. enterocolitica* strains listed are derivatives of AJD3.

**TABLE 2:** pIV mutants defective for induction of the Psp response

Isolate	Amino acid substitution(s)	Φ(*pspAp-lacZ*) activity (%)^a^
14	S28P N88D D225G S274C S276P V281E	10
	I294V G339D L409I S417P E419V	
34	L335Q, K381I	11
52	L188H, K381N, P396P, R424H	8
57	L373S, G399S	10
63	S18T S45T D100G D129D L199M L213S F249L L377H	10
69	M13I N128S V153A T355N	8
74	T42K D109G A303G	12
78	V56E A176T V238I T277R L278L P390L L392Q V416G K419R	8
104	K419I	12
115	L373S G399S	5
116	L85F V140D	6
117	F302Y V373A L426F	9
123	L222M G309A	9
124	P194M	4
132	K11N R78C G238C F411L S421N	8
181	N95I E150D L193L L276P	8
195	Q50R F255L LS356F	10

a The percentage of β-galactosidase activity in a strain with pBAD18-Kan encoding the mutant pIV, relative to wild-type pIV activity. Typically, a strain producing wild-type pIV had approximately 500 to 600 Miller units of β-galactosidase activity, whereas a strain with the empty pBAD18-Kan plasmid had approximately 30 Miller units of β-galactosidase activity.

By focusing on non-Psp-inducing pIV mutants that retained toxicity, we had hoped to favor the identification of secretins that still formed envelope-damaging multimers. Even so, when we analyzed unheated cell lysates by using anti-His_6_ immunoblotting, abundant SDS-resistant multimers were detected for wild-type pIV but not for any of the mutants (analysis of samples after boiling to dissociate any multimers showed that all of the mutants were made at a similar level as with the wild-type pIV [data not shown]). Therefore, even though we had attempted to bias the screen toward finding mutants that still multimerized, every mutant we isolated that was defective for induction of the Psp response was also partially or totally defective in forming SDS-resistant multimers. Definitive conclusions cannot be made from this failure to isolate a particular class of mutant in the screen. Nevertheless, these findings suggest that no single contact with a specific secretin amino acid is essential for induction of the Psp response, unless such an amino acid is also important for multimerization. However, these findings do provide independent support for the idea that the ability of secretins to form multimers is essential to induce the Psp response (25).

### Converting the pIV secretin to a nonmultimerizing form prevents its interaction with Psp proteins.

**FIG 6:**
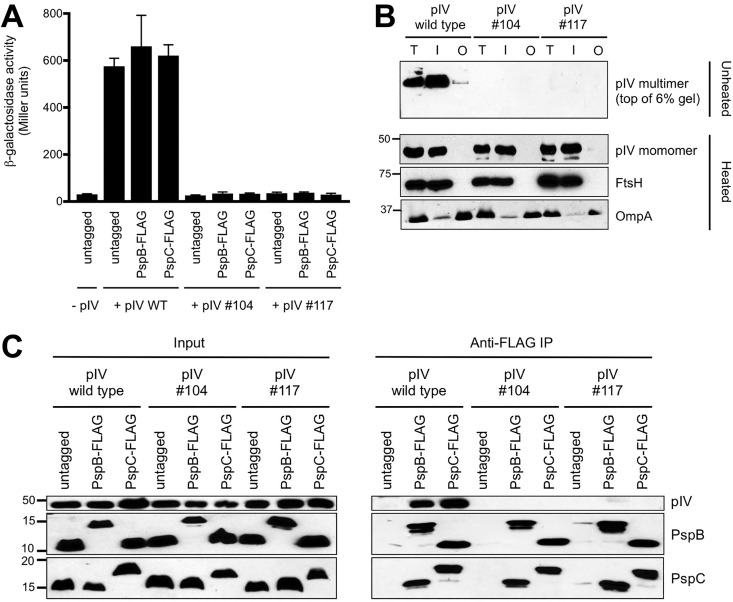
Conversion of pIV secretin to nonmultimerizing forms prevents its interaction with Psp proteins. (A) Φ(*pspA-lacZ*) operon fusion expression. Strains contained chromosomal *pspB*, *pspC*, or neither (untagged) modified to encode a C-terminal 3×FLAG epitope tag as indicated. Strains also contained empty *araBp* expression plasmid pBAD18-Kan (−pIV) or derivatives encoding pIV-His_6_ (+pIV WT) or the mutant derivatives 104 and 117 listed in Table 2. Error bars indicate the positive standard deviations from the means. (B) pIV subcellular localization and multimer detection. Anti-His_6_, anti-FtsH (inner membrane control), and anti-OmpA (outer membrane control) immunoblot analyses results are shown for subcellular fractions from the +pIV strains used in the experiment summarized in panel A. T, total membrane fraction; I, inner membrane fraction; O, outer membrane fraction. Approximate positions of molecular mass marker proteins (in kilodaltons) are indicated on the left. (D) Immunoblot analysis of input lysates and coimmunoprecipitates (anti-FLAG IP) derived from the +pIV strains used in the experiment summarized in panel A. PspB and PspC were detected with polyclonal antisera, and pIV was detected with anti-His_6_ monoclonal antibody. Approximate positions of molecular mass marker proteins (in kilodaltons) are indicated on the left.

Conversion of PilQ from a nonmultimerizing to a multimerizing form promoted its interaction with Psp proteins (Fig. 5). The non-Psp-inducing pIV mutants isolated in our screen allowed us to test the reverse idea: does conversion of pIV from a multimerizing to a nonmultimerizing form prevent its interaction with Psp proteins? This would further test the conclusion that multimerization is essential for secretins to complex with the Psp proteins. Therefore, we chose two pIV mutants, isolate 104 with a single K419I mutation and isolate 117 with F302Y, V373A, and L426F mutations (Table 2). In contrast to wild-type pIV, both mutants failed to induce Φ(*pspA-lacZ*) expression and did not produce detectable SDS-resistant multimers (Fig. 6A and B). However, they still localized to the IM fraction (Fig. 6B). To test if these nonmultimeric versions of pIV could form a complex with PspB and PspC, we used them as the prey in co-IP. Unlike wild-type pIV, neither mutant coimmunoprecipitated with PspB-FLAG or with PspC-FLAG (Fig. 6C). This further reinforced the conclusion that the Psp proteins interact with secretins only when they form multimers.

## DISCUSSION

**FIG 7:**
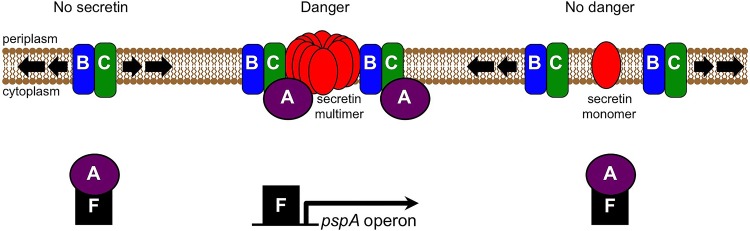
Model. In the absence of a mislocalized secretin, PspBC scan the membrane in their off state and PspA remains in an inhibitory complex with PspF. Mislocalization of a multimeric secretin into the inner membrane is a potentially dangerous condition that causes PspBC to switch to the on state, which leads to sequestration of PspA from PspF, so that PspF is free to induce *pspA* operon expression. PspBC (and PspA) also interact with the mislocalized secretin and prevent it from permeabilizing the inner membrane by an unknown mechanism. In contrast, when a mislocalized secretin cannot multimerize, there is no danger to inner membrane integrity, and so PspBC do not engage it and remain in their off state.

The Psp response has been studied for almost three decades, but important questions remain unanswered, including the precise inducing signal(s) and how the Psp effector proteins alleviate cell envelope stress (7, 8). The “phage shock” name arose because the Psp response was induced during filamentous phage infection of *E. coli*, which resulted from the phage-carried pIV secretin gene being mislocalized into the IM (10, 32). Subsequent studies revealed a surprisingly specific relationship between secretin mislocalization and the Psp response. Analyses in *E. coli*, *Salmonella enterica*, and *Y. enterocolitica* showed that secretin mislocalization induces *psp* gene expression without significantly affecting the expression of other genes (19, 20). A random screen of *Y. enterocolitica* mutants found that only Psp-defective strains were severely and specifically sensitive to secretin-induced stress (20). Furthermore, only PspB and PspC are required to prevent mislocalized secretins from causing lethal IM permeability in *Y. enterocolitica* (25). Here, we made a major advance in our understanding of the Psp system by discovering a molecular mechanism that offers an explanation for this remarkable specificity: the Psp effector proteins form a complex with mislocalized secretins in the IM. Our data also show that the Psp system can distinguish between multimeric and monomeric versions of the same secretin, perhaps engaging the dangerous multimers and negating their toxicity while ignoring nonthreatening monomers (Fig. 7).

The PspBC complex has two roles, which genetic analysis has shown to be independent (17, 24). First, it is a regulatory complex that responds to inducing cues such as secretin mislocalization by altering its physical arrangement, sequestering PspA away from PspF and activating the response (14, 15) (Fig. 7). Second, once the response is activated, the level of PspBC increases and they are required to prevent mislocalized secretins from permeabilizing the IM (25). Our discovery that PspBC are in a complex with a mislocalized secretin raises the question of whether this plays a role in their regulatory function (to detect the secretin), their effector function (to detoxify the secretin), both, or neither. We cannot yet answer this, but it might be addressed in the future by isolating PspBC or secretin mutants that interfere with the interaction and then testing the effects on secretin-dependent Psp induction and toxicity. We have not yet been able to identify this class of PspB or PspC mutants, and although our pIV mutagenesis screen identified noninteracting pIV mutants, all were deficient in multimerization. Regardless, we speculate that the interaction might be important to prevent secretin toxicity, because among the Psp proteins, only PspB and PspC are required to prevent secretin toxicity (20, 25). Furthermore, production of *Y. enterocolitica* PspBC prevented secretin toxicity in *Pseudomonas aeruginosa*, which lacks the Psp response (33). These observations are consistent with PspBC being able to prevent secretin toxicity directly, and a specific interaction is consistent with a direct mechanism. It is clear that multimeric secretins mislocalize into the IM even when PspBC are present (references 9, 25, and 34 and this work). Therefore, PspBC might complex with the mislocalized secretin to distort its conformation and collapse its pore, preventing IM permeabilization. Investigating this will not be trivial, but it might be amenable to future *in vitro* approaches.

There is also evidence that PspA functions as a stress-relieving effector of the Psp system. For example, when the Psp response is induced, PspA is the most abundant Psp protein in *E. coli*, and it is also the most highly conserved Psp protein (10, 35). Numerous studies have uncovered phenotypes consistent with PspA being important to maintain the proton motive force (34, 36–42). PspA was present in the PspBC-secretin complex, raising the possibility that PspA also plays a role in mitigating secretin stress (Fig. 1 and 3). However, we expected PspA to be present because it has been well established that it associates with PspBC when the Psp response is induced (14, 18). Nevertheless, our previous work unequivocally demonstrated that PspA is dispensable for secretin stress tolerance, whereas PspB and PspC are absolutely required (25). Therefore, although PspA is an important protein, it apparently does not play a role in preventing secretin-induced toxicity.

Aside from preventing secretin toxicity, it is also possible that PspBC-secretin complex formation is a direct mechanism by which PspBC detect a mislocalized secretin to activate the Psp response. Direct detection would provide the high level of specificity indicated by a mislocalized secretin inducing only the Psp response (19, 20). There is precedent for this, because direct detection of mislocalized proteins has been implicated in inducing the *E. coli* RpoE and Cpx extracytoplasmic stress responses (43, 44). However, without more experimentation, including isolation of the noninteracting mutants referred to above, we must be cautious about speculating that the interaction has any regulatory role. It is equally plausible that PspBC respond to a consequence of secretin mislocalization, such as a change in IM properties. A study with *E. coli* showed that PspA preferentially bound to phospholipid membranes *in vitro* with high stored curvature elastic (SCE) stress and that this disrupted a PspA-PspF complex (45). This suggests that SCE stress might be a Psp-inducing trigger, and certainly the insertion of large secretin multimers into the IM might affect SCE stress. However, the PspBC complex was not part of that *in vitro* experimental system, and there is no way to measure SCE stress *in vivo*. Regardless of whether SCE stress or some other property of the IM is a factor, the membrane disruption by multimeric secretins could be the trigger, rather than their direct interaction with Psp proteins.

We cannot yet conclude if secretins interact directly with PspABC and if so, with which Psp protein(s). Examination of the coimmunoprecipitates by SDS-PAGE and silver staining has not revealed any other abundant proteins in the complex (D. Srivastava and A. J. Darwin, unpublished data). Therefore, it is likely to be a direct interaction with one or more Psp proteins. However, attempts to test for secretin co-IP with individual Psp proteins have been inconclusive because of Psp protein instability and/or toxicity in the absence of others (25, 46) (Srivastava and Darwin, unpublished). Regardless of exactly where contact with the secretin might occur, it is interesting to consider what feature of a secretin determines it. An obvious possibility is a conserved sequence feature of the secretin. The sequences and domain structures of secretins vary considerably, but they all share a conserved C-terminal secretin domain which is involved in multimerization and forms the OM rings of the multimer (21). Therefore, the secretin domain might contain a recognition motif for the Psp system. However, our experiments suggest that recognition is a more complex process. PspBC did not coimmunoprecipitate with the PilQ secretin unless it was converted to the multimerizing form (Fig. 5). Furthermore, PspBC complex formation with the pIV secretin was abolished by different mutations that converted it to a nonmultimerizing form (Fig. 6). This strongly suggests that in order for a secretin to be recognized by the Psp system, and thus form a complex with Psp proteins, it must be a multimer. This does not rule out recognition of a conserved sequence motif, but it means that if such a motif exists, then multimerization is required for the correct stoichiometry or for presentation of the motif in the required context.

In summary, this work has shown that Psp effector proteins form a specific complex with mislocalized secretins in the IM. For the first time, these findings provide a satisfying mechanism to explain the remarkably specific relationship between secretins and the Psp system. Furthermore, our demonstration that complex formation occurs only if a secretin multimerizes suggests that the Psp system has evolved to engage secretins only when they pose a danger to IM integrity. Without the ability to multimerize, there is no potential for the secretin to cause lethal IM permeability, and it makes sense that the Psp proteins ignore these innocuous monomers. The challenges for the future will be to characterize these Psp-secretin complexes, perhaps with a combination of structural and other *in vitro* approaches, and also isolating and analyzing mutant Psp proteins that cannot interact with secretin multimers.

## MATERIALS AND METHODS

### Bacterial strains, plasmids, and growth conditions.

Bacterial strains and plasmids are listed in Table 1. The DNA sequences of all PCR-generated plasmid inserts were verified. Strains were grown in Luria-Bertani medium with antibiotics used at concentrations described previously (47).

### Plasmid constructions.

Plasmid pAJD2446 carrying the gene for PilQ-P562L-His_6_ was made by splicing by overlap extension (SOE) PCR (48). Briefly, the insert of pAJD1806 (Table 1) was amplified in two sections as separate PCR fragments with primers that generated a short overlap between them, which encoded the P562L mutation. These two fragments were joined by SOE PCR and cloned into pVLT35 as an EcoRI/SacI fragment. Plasmid pAJD2455, which carries the gene encoding YscC-His_6_, was made by amplifying the insert of pAJD126 (9) by using a downstream primer that added a region encoding His_6_ to the end of *yscC*, followed by a stop codon and a XhoI restriction site. This fragment was cloned into pVLT35 as a SacI/XhoI fragment. Plasmid pAJD2506 carrying the gene for pIV-His_6_ was made by amplifying the insert of pAJD1329 (20) by using an upstream primer that added a SacI restriction site followed by a strong ribosome binding site and a downstream primer that added a region encoding the sequence from His_6_ to the end of *gIV*, followed by a stop codon and an XbaI restriction site. This fragment was cloned into pBAD18-Kan as a SacI/XbaI fragment. Plasmid pAJD2657 carrying the gene for AcrZ-3×FLAG was made by amplifying *acrZ* from strain AJD3 genomic DNA by using an upstream primer that added a SacI restriction site followed by a strong ribosome binding site and a downstream primer that added a region encoding 3×FLAG to the end of *acrZ*, followed by a stop codon and an XbaI restriction site. This fragment was cloned into pAJD2144 as a SacI/XbaI fragment. Plasmid pAJD2779, carrying the gene for YsaC-3×FLAG, was made by amplifying *ysaC* from strain AJD3 genomic DNA by using an upstream primer that added a SacI restriction site and a downstream primer that added a region encoding 3×FLAG to the end of *ysaC*, followed by a stop codon and an XbaI restriction site. This fragment was cloned into pBAD33 as a SacI/XbaI fragment.

### Construction of *Y. enterocolitica* strains.

To make strains AJD4609 and AJD4740, which produced PspB-3×FLAG and PspC-FLAG, two ∼0.5-kb fragments surrounding the stop codon of *pspB* or *pspC* were amplified from the *Y. enterocolitica* chromosome. For each fragment, one of the primers incorporated a region encoding a 3×FLAG epitope. The fragments were then joined by SOE PCR via their overlapping 3×FLAG sequences to generate an ∼1.2-kb fragment with the region encoding 3×FLAG inserted immediately upstream of the *pspB* or *pspC* stop codon. These fragments were cloned into plasmid pRE112 and then used to fuse the 3×FLAG-encoding region to the native *psp* target gene by integration, selection for sucrose-resistant segregants, and confirmation by colony PCR and DNA sequencing of the PCR product.

### Coimmunoprecipitation.

Saturated cultures were diluted to an optical density at 600 nm (OD_600_) of approximately 0.08 in 300-ml LB broth with appropriate antibiotics in a 1-liter flask and grown in a rotary shaker at 250 rpm and 37°C. Secretin expression was induced after 3 h by adding 0.02% (wt/vol) arabinose (for YsaC-His_6_ and pIV-His_6_) or 100 µM isopropyl-β-d-thiogalactopyranoside (IPTG; for YscC-His_6_, PilQ-His_6_, and PilQ-P562L-His_6_). After a further 3 h, cells from the equivalent of 300 ml of culture at an OD_600_ of 1 were harvested by centrifugation. Cell pellets were suspended in 5 ml of 137 mM NaCl, 2.7 mM KCl, 4.3 mM Na_2_HPO_4_, 1.4 mM KH_2_PO_4_ (phosphate-buffered saline [PBS]) per g (wet weight) of pellet, and Roche Complete protease inhibitor cocktail was added at a 2× concentration. The cell suspension was frozen at −20°C, and then DNase I was added (1 ml of 1.67 mg/ml per 5 g [wet weight] of cells) prior to disrupting the cells by sonication. Unbroken cells were removed by centrifugation at 20,000 × *g* for 30 min, and then the supernatant was centrifuged at 100,000 × *g* for 2.5 h to isolate the membrane fraction. The membrane pellet was resuspended in 10 ml/g of nondenaturing lysis buffer (50 mM Tris-HCl [pH 7.5], 300 mM NaCl, 5 mM EDTA, and 2× Roche Complete protease inhibitor cocktail) and homogenized with a tissue grinder. One percent *n-*dodecyl-β**-**d-maltoside (DDM) was added, and the suspension was rocked at 4°C for 2 h to solubilize the membrane proteins. Insoluble material was removed by centrifugation at 14,000 × *g* for 30 min. The supernatant (input lysate) was precleared by incubation with 30 µl protein A-Sepharose beads for 30 min at 4°C, followed by removal of the protein A-Sepharose by centrifugation.

A protein A-Sepharose–anti-FLAG antibody complex was made by mixing 50% protein A-Sepharose slurry with anti-FLAG M2 monoclonal antibody (Sigma-Aldrich; 60 µl of 50% protein A-Sepharose slurry added to 1 µl of anti-FLAG antibody) and incubating for 3 h at 4°C with rocking. After washing, the FLAG-associated beads were resuspended in 60 µl nondenaturing lysis buffer and incubated with the precleared input lysate at 4°C overnight. The beads were isolated by centrifugation and washed twice with nondenaturing lysis buffer and then three times with PBS containing 0.1% (vol/vol) Triton X-100. The beads were resuspended in 60 µl SDS-PAGE sample buffer and boiled before analysis by immunoblotting.

### Subcellular fractionation.

Cell pellets were suspended in 4.9 ml of 200 mM Tris-HCl, 20 mM EDTA (pH 7.5) containing 2× Roche Complete protease inhibitor cocktail and then frozen at −20°C. The suspension was thawed and cells were lysed by sonication. Unbroken cells and other debris were removed by centrifugation at 16,000 × *g* for 4 min, and then the supernatant was centrifuged at 100,000 × *g* for 2 h to isolate the membrane fraction. To wash the membrane pellet, it was resuspended in 1 ml of 50 mM phosphate buffer (pH 7.5), 1 M NaCl (wash buffer) followed by centrifugation at 100,000 × *g* for 1 h. The membrane pellet was resuspended in 300 µl of 20 mM Tris-HCl (pH 7.5), and 50 µl was removed as the total membrane fraction sample. A 225-μl volume of 20 mM Tris-HCl, 0.9% (wt/vol) Sarkosyl, 2× Roche Complete protease inhibitor cocktail was added to the remaining total membrane fraction, and the mixture was rotated at room temperature for 30 min; 400 µl was centrifuged at 100,000 × *g* at 4°C for 1 h and 150 µl of the supernatant was retained as the IM fraction. The pellet was resuspended in 200 µl of 20 mM Tris-HCl (pH 7.5), 2% (wt/vol) SDS and retained as the OM fraction.

### pIV random mutagenesis.

The insert of pAJD2506 was amplified by PCR using the GeneMorph II random mutagenesis kit (Agilent Technologies) in five independent reaction mixtures, each containing ∼200 ng of plasmid DNA, 200 µM of each deoxynucleoside triphosphate, 300 nM each primer, and 2.5 units of Mutazyme II DNA polymerase. The cycling program was 30 cycles of 95°C for 30 s, 54°C for 30 s, and 72°C for 2 min. The products were digested with SacI and XbaI, ligated into pBAD18-Kan, and used to transform *E. coli* DH5α. Transformant colonies were combined and plasmid DNA was isolated, resulting in five independent mutant libraries. Each library was used to transform *Y. enterocolitica* strain AJD977 by electroporation, followed by screening for mutant phenotypes as described in the Results section.

### β-Galactosidase assays.

Saturated cultures were diluted into 5 ml of LB broth in 18-mm-diameter test tubes to an OD_600_ of approximately 0.04. The cultures were grown on a roller drum at 37°C for 2 h. Then, 0.02% (wt/vol) arabinose (for YsaC-His_6_ and pIV-His_6_) or 100 µM IPTG (for YscC-His_6_) or 300 µM IPTG (for PilQ-His_6_ and PilQ-P562L-His_6_) was added to induce secretin production. Cells were grown for another 2 h at 37°C prior to harvest. β-Galactosidase enzyme activity was determined at room temperature in permeabilized cells as described elsewhere (31). Activities are expressed in arbitrary Miller units (32). Individual cultures were assayed in duplicate, and average values from three independent cultures are reported.

### Growth curves.

Saturated cultures were diluted into 5 ml of LB broth containing 300 µM IPTG to induce PilQ-His_6_ or PilQ-P562L-His_6_ production in 18-mm-diameter test tubes so that the initial OD_600_ was approximately 0.1. The cultures were grown on a roller drum at 37°C for 8 h, and 0.1-ml samples were removed at hourly intervals for OD_600_ measurements.

### Detection of secretin monomers and SDS-resistant multimers.

To detect secretin multimers, unheated samples in SDS-PAGE sample buffer were separated by SDS-PAGE on a gel containing 6% polyacrylamide and then transferred to a nitrocellulose membrane by using a Bio-Rad Trans-Blot SD semidry transfer cell (25 V for 1.5 h). To detect secretin monomers, equivalent samples were boiled for 10 min prior to separation by SDS-PAGE on a gel containing 12.5% polyacrylamide. In both cases, secretin proteins were detected with antibodies as described below.

### Immunoblotting.

Samples were separated by SDS-PAGE and transferred to a nitrocellulose membrane by semidry electroblotting. Equal loading and transfer were confirmed by Ponceau S staining (Amresco). Enhanced chemiluminescence detection followed sequential incubation with a diluted polyclonal antiserum or monoclonal antibody, and then goat anti-rabbit IgG or goat anti-mouse IgG horseradish peroxidase conjugate (Sigma) was used at the manufacturer’s recommended concentrations. Dilutions of polyclonal antisera were 1 in 10,000 for anti-FtsH and anti-PspA (27), anti-PspC (24), and anti-OmpA (Antibody Research Corp.) and 1 in 20,000 for anti-PspB (17). Dilutions of monoclonal antibodies were 1 in 5,000 for both anti-FLAG (Sigma) and anti-His_6_ (GenScript).
